# Advances in Colorectal Cancer

**DOI:** 10.1155/2016/7347648

**Published:** 2016-11-06

**Authors:** Nikolaos I. Nikiteas, Markus Weber, Anthony Antoniou, Dimitrios Dimitroulis

**Affiliations:** ^1^Medical School, National and Kapodistrian University of Athens, Athens, Greece; ^2^Stadtspital Triemli, Zürich, Switzerland; ^3^Imperial College London, London, UK

Colorectal cancer (CRC) is one of the leading causes of cancer-related death worldwide. Surgery remains the cornerstone for curative treatment, and operative techniques have changed considerably over the past decades. Laparoscopic approach has become standard for colon cancer, and several developments including robotic-assisted surgery are evolving for rectal cancer. The addition of targeted therapies has prolonged life in metastatic disease to considerable extent. The genetic understanding of the disease has led to introduction of molecular classification proposals, which exemplifies knowledge translated from basic science to clinical care. Furthermore, the advance of currently available diagnostic modalities and the experimental investigation of novel technologies such as the implementation of nanoparticles brings considerable promise for the early detection, staging, and treatment of CRC and calls for improved stratification and prognostication to better allocate resources and justify associated costs, while reducing morbidity and even mortality from the disease.

A new wave of trial data, in the coming years, and an evolving knowledge of relevant biomarkers may bring us closer to new more effective screening and surveillance strategies. To best apply these insights, a number of important research questions need to be addressed, and new decision making tools must be developed. To promote the basic and clinical research field in CRC, we invited investigators from around the globe to contribute original research articles, as well as review articles that will stimulate continuing efforts to expand the diagnosis and treatment of CRC.

In this current special issue, we focus on recent advances in the field of novel targets and molecular interventions, which might help reveal the possible mechanism of tumorigenesis, progression, metastasis, and recurrence and contribute to emerging therapeutics of colorectal cancer.

D. Rodrigues et al. in their article titled “Predictive Biomarkers in Colorectal Cancer: From the Single Therapeutic Target to a Plethora of Options” review CRC genetic markers that could be useful in predicting the sensitivity/resistance to chemotherapy. Most recently, biologic agents have been proven to have therapeutic benefits in metastatic CRC alone or in association with standard chemotherapy. However, patients present different treatment responses, in terms of efficacy and toxicity; therefore, it is important to identify biological markers that can predict the response to therapy and help in selecting patients that would benefit from specific regimens.

S. Garritano et al. analysed perioperative and oncological outcomes of minimally invasive simultaneous resection of primary colorectal neoplasm with synchronous liver metastases. The overall morbidity and mortality rate were 18% and 1.3%, respectively. The most common complication was colorectal anastomotic leakage. Data concerning oncologic outcomes were too heterogeneous in order to gather definitive results. Although no prospective randomized trials are available, one-stage minimally invasive approach seemed to show advantages over conventional surgery in terms of postoperative short-term course.

J.-W. Oh et al. evaluated the value of Gadoxetic acid-enhanced liver MRI in the preoperative staging of colorectal cancer and estimated the clinical impact of liver MRI in the management plan of liver metastasis. Gadoxetic acid-enhanced liver MRI detected more metastatic nodules compared with PET/CT, especially for small (<2 cm) nodules. The newly detected nodules induced management plan change in 43.8% (7/16) of patients.

K. Walkiewicz et al. showed that various genes, especially ADAM17, MMP9, EphA2, TIMP1, ICAM 11, and CD4, may be used as prognostic markers of advanced stages of colorectal cancer, contributing to the development of new lines of therapy focused on reducing metastasis of the primary tumor.

Finally, this special issue includes several intriguing achievements in the area of novel targets in treatment of unresectable metastatic colorectal cancer as summarized by S. Lee and S. C. Oh, as well as a cost-effectiveness assessment study of immunochemical tests in mass colorectal cancer screening performed by S.-R. Cai et al.

